# Supplementary material for: The Biochemical Alteration of Enzymatically Hydrolysed and Spontaneously Fermented Oat Flour and Its Impact on Pathogenic Bacteria
Source: Foods. 2022 Jul 12;11(14):2055. doi: 10.3390/foods11142055 (PMC9316710; doi:10.3390/foods11142055)
Supplement: Supplementary file 1 [file foods-11-02055-s001.zip › foods-1771149-supplementary.pdf]

Supplementary materials:

The Biochemical Alteration of Enzymatically Hydrolysed and Spontaneously Fermented Oat Flour and Its Impact on Pathogenic Bacteria

Paulina Streimikyte [1], Jurgita Kailiuviene [2], Edita Mazoniene [2], Viktorija Puzeryte [1], Dalia Urbonaviciene [1], Aiste Balciunaitiene [1], Theodore Daniel Liapman [3], Zygimantas Laureckas [4], Pranas Viskelis [1] and Jonas Viskelis [1,*]

Commercial oat-based beverages samples for PCA analysis using electronic tongue αAstree (Alpha M.O.S., Toulouse, France).

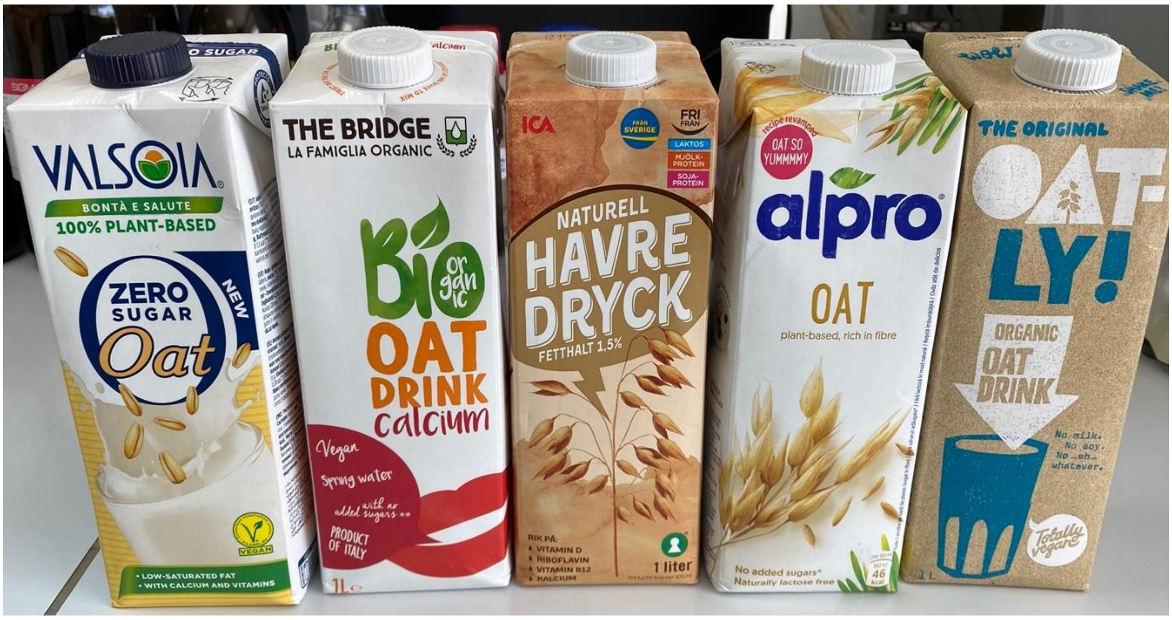

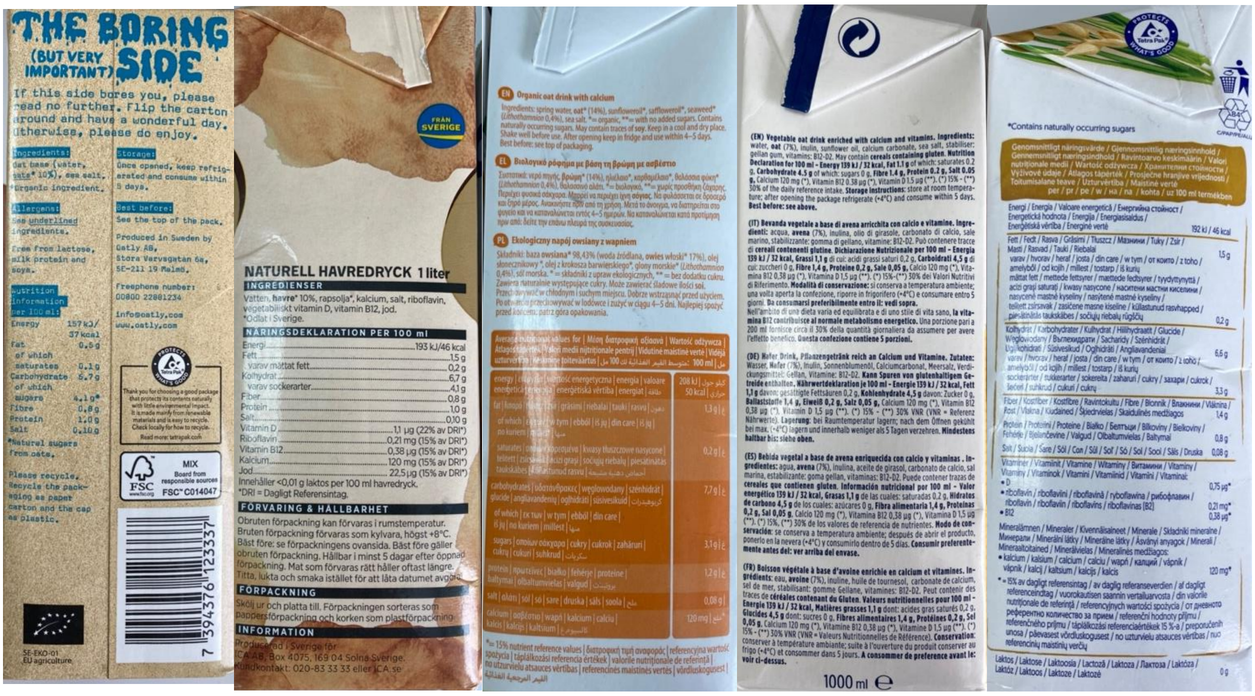

Commercial OBD_1 | Commercial OBD_2 | Commercial OBD_3 | Commercial OBD_4 | Commercial OBD_5

Figure S1. Samples are named in accordance to research Figure 5. Where **6** – Commercial OBD_1 “Oatly”; **7** – Commercial OBD_2 “Naturell Havre Dryck”; **8** – Commercial OBD_3 “Bio Organic Oat Drink”; **9** – Commercial OBD_4 “Valsoia”; **10** – Commercial OBD_5 “Alpro”.
